# Electrical Properties of Lumbar Paraspinal Muscles in Young Adults With and Without Chronic Low Back Pain Based on Electrical Impedance Myography: A Cross-Sectional Study

**DOI:** 10.3389/fneur.2021.789589

**Published:** 2022-02-17

**Authors:** Hongjiang Wang, Jiaxuan Zheng, Qiuhua Yu, Ziyan Fan, Wai Leung Ambrose Lo, Le Li, Chuhuai Wang

**Affiliations:** ^1^Department of Rehabilitation Medicine, The First Affiliated Hospital, Sun Yat-sen University, Guangzhou, China; ^2^Institute of Medical Research, Northwestern Polytechnical University, Xi'an, China

**Keywords:** electrical impedance myography, lumbar paraspinal muscle, chronic low back pain, electrical property, Roland–Morris Disability Questionnaire, Oswestry Disability Index

## Abstract

**Background:**

Lumbar paraspinal muscle (LPM) is important in spinal stabilization in patients with chronic low back pain (CLBP). However, the electrical properties of LPM in patients with CLBP remain unclear. Electrical impedance myography (EIM) is a novel and non-invasive technique that provides a simple quantitative evaluation of electrical properties of the LPM.

**Purpose:**

This study aimed to apply EIM to assess the electrical properties of the LPM between patients with CLBP and healthy control (HC).

**Methods:**

Thirty participants (15 CLBP participants; 15 healthy controls) were enrolled in the study. Participants in the CLBP group were asked to complete the visual analog scale (VAS), Oswestry Disability Index (ODI), and Roland–Morris Disability Questionnaire (RDQ) to assess the pain intensity and disability in daily life. Independent sample *t*-tests were adopted to analyze the basic characteristics between the two groups. At 5, 50, 100, and 200 kHz current frequencies, the electrical properties were measured on each side of the LPM. The EIM parameters of resistance (R), reactance (X), phase angle (PA), and Z value were analyzed by one-way analysis of variance (ANOVA), with age as covariate. Spearman's rank correlation coefficient analysis was applied to explore the relationships between the questionnaires and the EIM parameters.

**Results:**

The R and Z values of bilateral LPM in the CLBP group were significantly larger than those in the HC group; the PA decreased and the X did not change at these four tested current frequencies. At 5 kHz, Z and R on the right side were non-significantly different between patients and HCs. Correlation analysis showed that at 50 kHz, ODI and RDQ scores correlated negatively with the R of the bilateral LPM (*r* = 0.523, *r* = 0.581, respectively; *p* < 0.05). RDQ scores correlated positively with the PA of the right LPM (*r* = 0.521, *p* < 0.05).

**Conclusion:**

The electrical properties of the bilateral LPM differed between CLBP participants and healthy individuals, regardless of the different frequencies used. These altered electrical properties of the LPM in the patients with CLBP correlated to some extent with disability in daily life.

## Introduction

Chronic low back pain (CLBP) is a multifactorial disease with a lifetime prevalence of 75–84% in developed countries worldwide (1). It occurs in all age groups (2). CLBP brings great inconvenience and economic burden and affects individuals' activities of daily life. Annually, a large number of patients quit working because of CLBP (3). Despite the burden imposed by this disease, the pathogenesis of CLBP remains controversial (4). Panjabi supposed the mechanism of CLBP correlated with the neutral zone and instability mechanism (5). Disturbances in the stabilizing mechanism lead to spinal segments moving outside of their normal range of motion (the so-called neutral zone), causing tissue injury and initiating LBP (6). Since Panjabi first suggested this instability hypothesis of low back pain, the trunk muscles in patients with CLBP have become a research focus.

The lumbar paraspinal muscles (LPMs), such as the erector spinae and lumbar multifidus, are considered important for trunk-core stability (5). Many studies have reported that the structures and activation functions of these muscles may be altered in the patients with CLBP. The lumbar multifidus in these patients was found to be impaired, as its thickness and cross-sectional area (CSA) became smaller than those of healthy individuals (7, 8). Moreover, older adults with LBP were reported to have greater average multifidus muscle-to-fat indices and smaller average relative erector spinae muscle CSAs than did control participants without LBP (9). Using proton magnetic resonance spectroscopy, Mengiardi et al. (10) reported that significantly, more intramuscular fat was observed in the lumbar multifidus of young adults with CLBP than in those with healthy controls (HCs). Additionally, Wan et al. applied magnetic resonance imaging (MRI) to assess patients with acute and chronic LBP. They found that these patients were undergoing atrophy and fatty infiltration in the LPM (11). Although there have been many reports about changes in the LPM of patients with CLBP, the specific characteristics of the structural and organizational changes remain unclear.

Electrical properties are important characteristics of skeletal muscle. These properties involve the variation in impedance values when the electrical current flows along as opposed to across the muscle fibers (12). Electrical impedance myography (EIM) is an evolution of bioelectrical impedance analysis, which relies on the application and measurement of high-frequency and low-intensity electrical currents. It is widely used as a non-invasive method of body composition assessment of neuromuscular diseases (13), such as body myositis (14), Duchenne muscular dystrophy (15), amyotrophic lateral sclerosis (16), subacute stroke (17), and facioscapulohumeral muscular dystrophy (18), etc. EIM has several advantages that it is reliable, highly reproducible, and easy to operate (19, 20). In contrast to conventional needle electromyography and most standard neurophysiological techniques, EIM does not focus on measuring the inherent electrical activity of tissues. Instead, measurements are taken when energy is applied to the body, and the resultant surface patterns are analyzed over a small area of interest. Unlike ultrasound, in which energy is in the form of sound waves and the main output is in the form of image reconstruction, EIM uses an electrical current and the output comprises a set of quantitative parameters describing the state of the muscle, with few emphasis on imaging (13). The changes in electrical current flow reflect the properties of the tissue and thus can reveal information about tissue health or pathology. In terms of EIM parameters, the cell membranes (in the case of muscle, the sarcolemma) represent the capacitor, whereas the extra- and intracellular resistance (R) represent the resistors (13). The phase angle (PA) refers to the relationship between the two vector components, that is, R and reactance (X), of the impedance (21). Previous studies have demonstrated that the PA decreases in generalized neuromuscular diseases, and also in more focal disorders (22–24). On the other hand, the composition and architecture of the muscle changes affect the impedance values and their frequency dependency (13). The impedance characteristics are related to the underlying structure and properties of the muscle with varying frequency (25). Using a spectrum of frequencies would provide a richer assessment of muscle tissue than using a single frequency. Evaluating EIM parameters under different current frequency conditions would contribute to the assessment of muscle tissue status and to finding possible pathological changes in muscle composition (25).

Since EIM has been widely applied to the study of muscular disorders (12), we considered that EIM may be a novel way to demonstrate pathological characteristics of the LPM in patients with CLBP. However, few is known about the electrical properties of the LPM in patients with CLBP at present. Whereas a previous study had characterized of the electrical properties of the LPM in patients with acute LBP (21), there has been no such study in CLBP. Nonetheless, this previous study on acute LBP introduced a guide for LPM assessment in patients with CLBP (21). In that study, the intracellular R was found to be higher in patients with CLBP than in HCs. The authors considered that the electrical properties of the LPM in patients with CLBP may differ from those of HCs. If this hypothesis was proven true, it would contribute to an understanding of the pathogenesis of CLBP and might offer a novel diagnostic method for CLBP, or an objective means of assessment of the disease status (13).

Thus, to verify the above hypothesis, we here explored the electrical properties of the LPM in patients with CLBP and in HCs using a multifrequency EIM device. To investigate the roles of electrical properties of the LPM in the disability of patients with CLBP, we performed a correlation analysis between CLBP-related questionnaire scores and the electrical parameters of the LPM in these patients.

## Materials and Methods

This cross-sectional study compared differences in the electrical properties of the LPM between patients with CLBP and healthy young adults. To explore the electrical conductivity of the muscle fibers further, we measured the electrical properties of the LPM bilaterally under four different current frequency conditions (5, 50, 100, and 200 kHz). Moreover, the relationships between the EIM parameters assessed at a current frequency of 50 kHz and the functional index of daily life, based on the Oswestry Disability Index (ODI) and the Roland–Morris Disability Questionnaire (RDQ) scores, were analyzed.

### Participants

Thirty individuals (*n* = 15 in each of the CLBP and HC groups) were recruited to participate in this study through advertisements on social platforms and at outpatient services. The inclusion criteria of the CLBP group were a medical diagnosis of non-specific LBP with pain and symptoms persisting for >3 months; pain quantified at >3 cm on the visual analog scale (VAS), for which medical treatment had been sought; age 18–40 years; and no history of other diseases. Participants were excluded if they were not aged 18–40 years or had LBP of traumatic or structural origin, LBP with neurological symptoms, or pain radiating to the lower leg(s), previous back surgery, spinal tumors or infections, or neurological and/or musculoskeletal disorders unrelated to LBP (26). The diagnosis of CLBP was based on the diagnostic guidelines published by the American College of Physicians and the American Pain Society (27).

The inclusion criteria for the HC group were the absence of LBP in the preceding 2 years and no history of other diseases (28). The exclusion criteria for HCs were age outside of the specified range, pregnancy even if the person who matched the inclusion criteria, or history of other diseases or surgery.

The study was approved by the Human Subjects Ethics Subcommittee of the first affiliated hospital (Registration Number: 2020425) and was performed in adherence to the tenets of the Declaration of Helsinki. The study had been recorded in Chinese Clinical Trial Registry (Registration Number: ChiCTR2100043113). All participants were enrolled in the study after obtaining written informed consent.

### Questionnaire Assessment

The degree of pain was quantified using a VAS, comprising a 10-cm scale with scores ranging from 0 (no pain) to 10 (severe pain). Participants in the CLBP group were instructed to complete the ODI (29) and RDQ (30) for the assessment of their pain-related disability and dysfunction in daily life. The ODI comprised 10 items on pain and activities of daily living (pain intensity, lifting, walking, social life, personal care, sitting, standing, sleeping, sex life, and traveling), and each item was scored from 0 to 5. The total score was calculated by summing the individual item scores. A higher total score indicated greater disability. The RDQ score was also calculated by adding the scores of the 24 items in the questionnaire. Each question had a response of “yes” (1 point) or “no” (0 points), with the total scores ranging from 0 (no disability) to 24 (maximum disability).

### EIM Data Collection

A multifrequency body composition analysis system (Imp SFB7, Inc., Sydney, NSW, Australia) was used to measure the electrical impedance of the participants' LPMs. This medical multifrequency device is capable of applying currents simultaneously at various frequencies from 2 kHz to 1 MHz. Two systematically trained rehabilitative clinicians were involved in data collection but were blinded to the participants' groups. The electrode arrangement for LPM testing referred to the principles described by Rutkove (13) and Hu et al. (17) and followed the standardized measurement methods of the Imp SFB7 for measuring the electrical properties of the LPM (31, 32). The Imp SFB7 is a linear EIM device, which was different from the device used in Ching et al.'s study (21).

**Figure 1:**
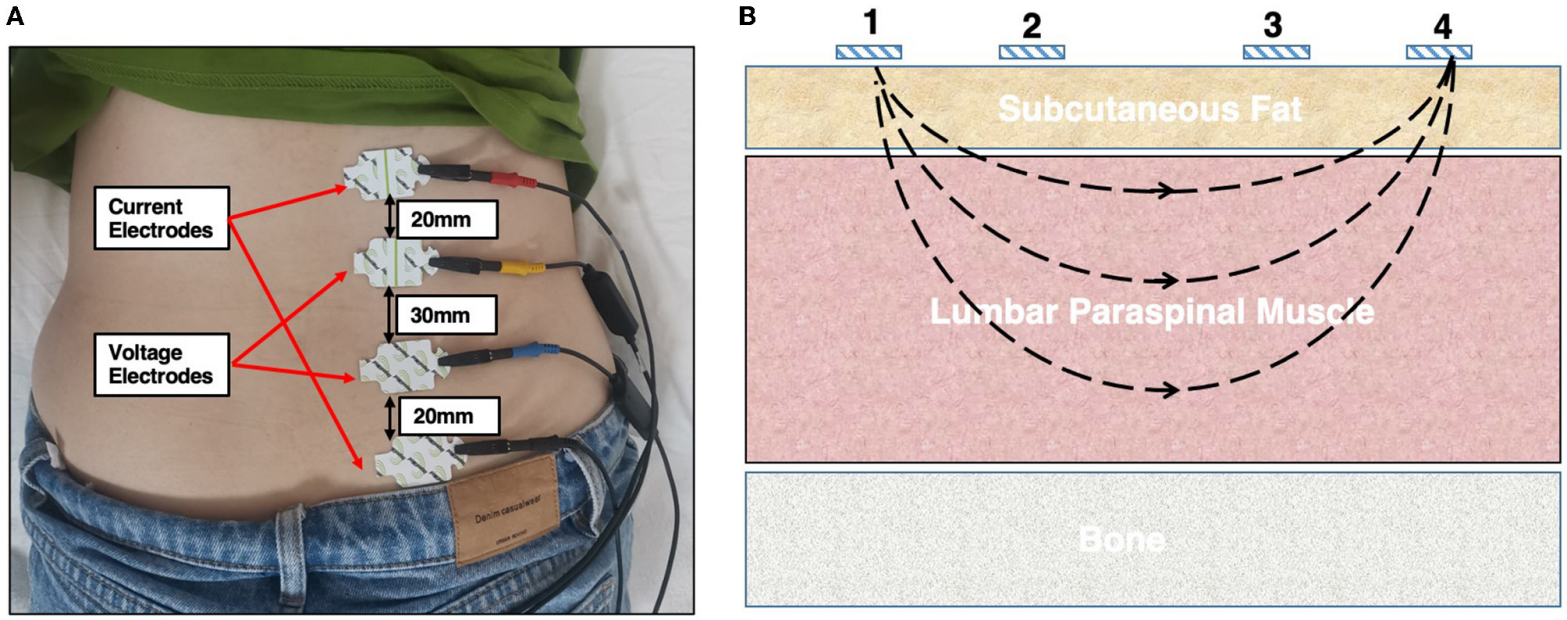
**(A)** Schematic diagram assessing the electrical properties of the lumbar paraspinal muscles using the EIM technique. A female participant was instructed to lie in a relaxed, prone position on the instrumented bed. One pair of current electrodes (red and black) and one pair of voltage electrodes (yellow and blue) were linearly arranged along the surfaces of the right LPM on the muscle belly. **(B)** Illustration of the positions of the four electrodes, where 1 and 4 are the current electrodes and 2 and 3 are the voltage electrodes. LPM, lumbar paraspinal muscles.

The participants were instructed to lie in the prone position with a soft pillow placed below the abdomen to keep the lumbar arch straight; thus, fully exposing the lower back. Before testing, the skin covering the LPM area was cleaned with 75% alcohol and was left to dry thoroughly. On each side, the upper edge of the third electrode was placed on the skin, 2 cm adjacent to the spinal process, and at the horizontal level of the spinal process to the anterior superior iliac crest. In the longitudinal direction, the 1st, 2nd, 3rd, and 4th electrodes (1st and 4th are current electrodes and 2nd and 3rd are voltage electrodes) were arranged vertically along the ridge process and were separated by 20, 30, and 20 mm, respectively (Figure 1A). The phase angle (PA), resistance (R), reactance (X) and Z values were recorded at 5, 50, 100, and 200 kHz. The contralateral LPM was similarly assessed (Figure 1B). The model of the electrode plates was L-DEX (Lymphedema Dual Tab Electrode, Pinkenba QLD 4008, Australia). To avoid possible bias, the parameters were measured three times in each participant. The operators were two physiotherapists with more than 3 years of clinical experience and were blinded to the participants' groups.

### EIM Parameter Processing

Impedance can be described mathematically. The EIM parameters commonly comprise R, X, PA, and Z. X consists of a combination of the two forms of reactance, capacitive reactance X_C_, and inductive reactance X_L_. The PA is calculated *via* standard trigonometric relationships, as follows (12):

(1) $$\begin{array}{r} PA\;=\;arctan\;\left(X/R\right) \end{array}$$

Z represents the complex impedance of the circuit, which is a combination of its inherent R and its X. The latter, produced by the presence of capacitors and/or inductors in the circuit, obstructs the current flow (12). Z is calculated as follows:

(2) $$\begin{array}{r} Z=\sqrt{R^{2}+{\left(XC-XL\right)}^{2}} \end{array}$$

In medical applications, this equation can be simplified by ignoring the X_L_ term, because inductance is thought to play a minimal role in standard bioimpedance measurements, and the X_C_ term can be simply written as X (12). In this study, all these parameters were processed using the ImpediMed SFB7 multifrequency analysis system.

### Statistical Analysis

Statistical analysis of the parameters was performed using SPSS version 26.0 (IBM Statistics, New York, NY, USA). The Kolmogorov–Smirnov test was used to determine normality of data distribution. The results showed that parameters in the two groups were normally distributed (the Kolmogorov–Smirnov test; *p* > 0.05), except for age and body mass index in the HC group, and RDQ and X at 5 kHz in the CLBP group. Independent sample *t*-tests were used to compare the basic characteristics of the two groups, and the chi-square test was used to compare sex. As age was significantly different between the CLBP and HC groups (Table 1), it was regarded as a covariate in the analysis of variance (ANOVA). One-way ANOVA with age as covariate was used to compare R, X, Z, and PA between the bilateral LPM in the two groups at each current frequency. Spearman's rank correlation coefficient was used to assess the correlation between the functional indexes and EIM parameters. Two-tailed *p*-values < 0.05 were considered statistically significant.

**Table 1:** Demographics of subjects in CLBP and HC groups (mean± SD).

	**CLBP**	**HC**	**t**	***P***
Sex^#^	Male 3, Female 12	Male 6, Female 9	NA	0.247
Age^&^	26.53 ± 2.70	23.20 ± 2.76	3.348	0.002^*^
Height^&^	164.80 ± 6.34	164.73 ± 7.30	0.027	0.979
Weight^&^	57.10 ± 8.88	56.67 ± 10.69	0.121	0.905
BMI^&^	20.94 ± 2.27	20.72 ± 2.48	0.249	0.805
VAS	5.19 ± 1.11	NA		
ODI	8.00 ± 4.33	NA		
RDQ	3.53 ± 2.53	NA		

*BMI, body mass index; VAS, visual analog scale; ODI, Oswestry Disability Index; RDQ, Roland–Morris Disability Questionnaire; NA, not application.*

# *chi-square test;*

& *independent sample t-tests*.

p-value marked with * means significant difference compared to the control group p < 0.05.

## Results

### Characteristics of Participants in the CLBP Group and HC Group

This study included 30 participants (15 in each of the CLBP and HC groups). There was no statistically significant difference in sex, height, weight, and body mass index between the two groups (*p* = 0.247, *p* = 0.979, *p* = 0.905, and *p* = 0.805, respectively). However, the average age differed between groups (*p* = 0.002). The baseline characteristics of the two groups are shown in Table 1.

### EIM Parameters in the CLBP Group and HC Group

Since the participants' ages differed between the two groups, it was regarded as a covariate factor in the analysis of variance used to calculate the electrical impedance parameters. We found that the R values of the left LPM were higher in the CLBP group than in the HC group at the current frequencies of 5 (*p* = 0.047), 50 kHz (*p* = 0.019), 100 (*p* = 0.017), and 200 kHz (*p* = 0.016). Furthermore, those of the right LPM differed between the two groups at 50 (*p* = 0.047), 100 (*p* = 0.043), and 200 (*p* = 0.044), but not at 5 kHz (*p* = 0.084).

The X values of the bilateral LPM showed no significant difference between the CLBP and HC groups at any of the four current frequencies (*p* > 0.05 for all).

The PA values of the bilateral LPM were lower in the CLBP group than in the HC group at 5 (left, *p* < 0.001; right, *p* = 0.005), 50 (left, *p* = 0.003; right, *p* = 0.007), 100 (left, *p* = 0.019; right, *p* = 0.015), and 200 kHz (left, *p* < 0.001; right, *p* = 0.024).

**Figure 2:**
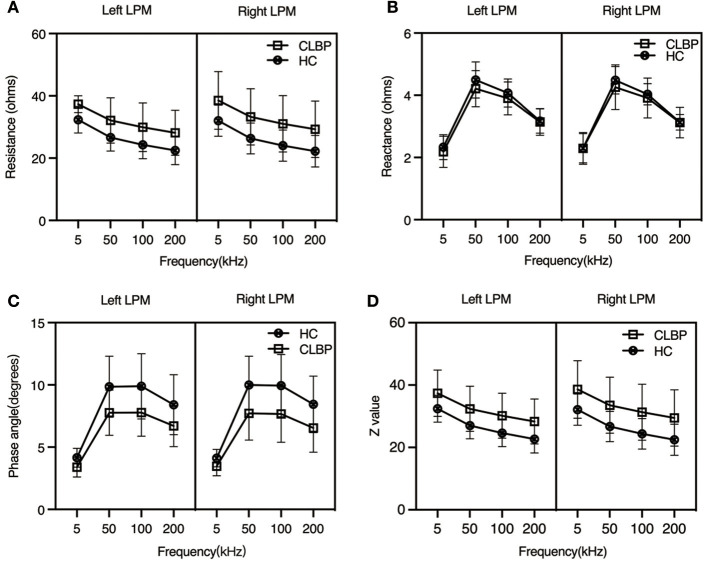
Impedance parameters from the bilateral lumbar paraspinal muscles of the chronic low back pain and healthy control groups at different current frequencies. At the four different current frequencies: **(A)** Resistance: mean R_CLBP_ > mean R_HC_; *p* < 0.05, except for the right side at 5 kHz (*p* = 0.084); **(B)** Reactance: mean X_CLBP_ < mean X_HC_; *p* > 0.05; **(C)** Phase angle: mean PA_CLBP_ > mean PA_HC_; *p* < 0.05; **(D)** Z value: mean Z_CLBP_ > mean Z_HC_; *p* < 0.05, except for the right side at 5 kHz (*p* = 0.085). CLBP, chronic low back pain; HCs, healthy controls.

The trend of the Z values was similar to that of the R values. The average Z values of the bilateral LPM were higher in the CLBP group than in the HC group at 50 (left, *p* = 0.020; right, *p* = 0.050), 100 (left, *p* = 0.017; right, *p* = 0.044), and 200 kHz (left, *p* = 0.017; right, *p* = 0.045), as was the Z value of the left LPM at 5 kHz (*p* = 0.048). However, this was not the case on the right side at 5 kHz (*p* = 0.085). The details are shown in Table 2 and are visually represented in Figure 2.

**Table 2:** EIM parameters of bilateral LPM in the groups at different frequencies (mean ± SD).

	**Group**	**5 kHZ**	**50 kHZ**	**100 kHZ**	**200 kHZ**
*R* value	CLBP-left LPM	37.31 ± 2.70	32.11 ± 7.29	29.91 ± 7.97	28.15 ± 7.22
	HC-left LPM	32.34 ± 4.28	26.62 ± 4.35	24.28 ± 4.47	22.46 ± 4.55
	*P*	0.047^*^	0.019^*^	0.017^*^	0.016^*^
	partial eta^2^	0.139	0.188	0.195	0.195
	CLBP-right LPM	38.53 ± 9.24	33.25 ± 9.03	31.03 ± 9.06	29.29 ± 9.05
	HC-right LPM	32.04 ± 4.99	26.34 ± 4.94	24.01 ± 4.99	22.24 ± 5.05
	*P*	0.084	0.047^*^	0.043^*^	0.044^*^
	Partial eta^2^	0.107	0.138	0.143	0.142
*X* value	CLBP-left LPM	2.18 ± 0.50	4.21 ± 0.58	3.90 ± 0.53	3.14 ± 0.43
	HC-left LPM	2.33 ± 0.40	4.49 ± 0.58	4.07 ± 0.45	3.17 ± 0.39
	*P*	0.053	0.089	0.336	0.923
	Partial eta^2^	0.131	0.103	0.034	0.000
	CLBP-right LPM	2.29 ± 0.51	4.26 ± 0.72	3.91 ± 0.64	3.12 ± 0.49
	HC-right LPM	2.30 ± 0.47	4.48 ± 0.44	4.03 ± 0.34	3.13 ± 0.25
	*P*	0.346	0.163	0.447	0.978
	partial eta^2^	0.033	0.071	0.022	0.000
PA	CLBP-left LPM	3.39 ± 0.78	7.77 ± 1.81	7.78 ± 1.90	6.70 ± 1.66
	HC-left LPM	4.15 ± 0.76	9.86 ± 2.45	9.89 ± 2.62	8.41 ± 2.41
	*P*	<0.001^**^	0.003^**^	0.009^**^	0.019^*^
	Partial eta^2^	0.420	0.277	0.229	0.186
	CLBP-right LPM	3.47 ± 0.77	7.72 ± 2.15	7.67 ± 2.28	6.54 ± 1.94
	HC-right LPM	4.12 ± 0.70	9.99 ± 2.32	9.95 ± 2.50	8.44 ± 2.27
	*P*	0.005^**^	0.007^**^	0.015^*^	0.024^*^
	Partial eta^2^	0.260	0.242	0.201	0.175
*Z* value	CLBP-left LPM	37.38 ± 7.43	32.40 ± 7.24	30.18 ± 7.22	28.34 ± 7.19
	HC-left LPM	32.43 ± 4.28	27.02 ± 4.26	24.64 ± 4.37	22.70 ± 4.48
	*P*	0.048^*^	0.020^*^	0.017^*^	0.017^*^
	partial eta^2^	0.139	0.183	0.192	0.194
	CLBP-right LPM	38.60 ± 9.24	33.54 ± 8.97	31.30 ± 9.00	29.45 ± 9.01
	HC-right LPM	32.13 ± 4.99	26.74 ± 4.85	24.36 ± 4.90	22.47 ± 4.98
	*P*	0.085	0.050^*^	0.044^*^	0.045^*^
	Partial eta^2^	0.106	0.135	0.141	0.141

*LPM, lumbar paraspinal muscle; X, reactance; R, resistance; PA, phase angle;*

p-value marked with * means p < 0.05,

p-value marked with ** means p < 0.01.

### Correlation Analysis Between the Parameters

We calculated the correlation of the VAS, ODI, and RDQ scores with R, X, and PA in the CLBP group. The Kolmogorov–Smirnov test showed that the VAS, ODI, and EIM data of each side of the LPM at 50 kHz were normally distributed (*p* > 0.05 for each parameter), but the RDQ scores were not (*p* < 0.05). Because VAS, ODI, and RDQ are discontinuous variables, Spearman's rank correlation coefficient was applied in the correlation analysis.

**Figure 3:**
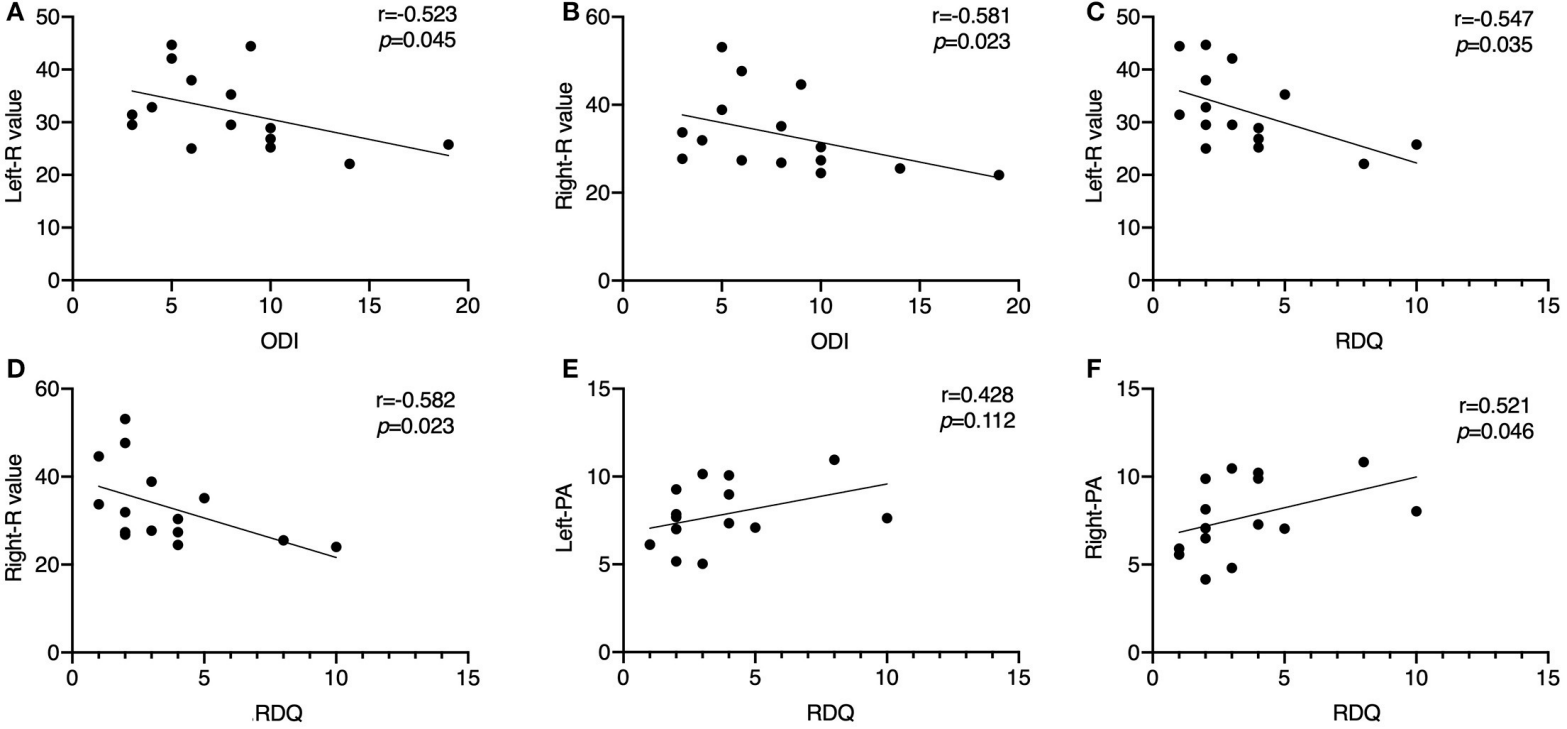
Scatter diagrams illustrating the correlation between Oswestry Disability Index score, Roland–Morris Disability Questionnaire score, resistance value, and PA of the unilateral lumbar paraspinal muscles in the chronic low back pain group at 50 kHz. **(A)** ODI score shows a negative correlation with the R value of the left LPM (*p* < 0.05); **(B)** ODI score shows a negative correlation with the R value of the right LPM (*p* < 0.05); **(C)** RDQ score shows a negative correlation with the R value of the left LPM (*p* < 0.05); **(D)** RDQ score shows a negative correlation with the R value of the right LPM (*p* < 0.05); **(E)** RDQ score has no significant correlation with the PA of the left LPM (*p* > 0.05); **(F)** RDQ score shows a positive correlation with the R value of the right LPM (*p* < 0.05). ODI, Oswestry Disability Index; R, resistance; LPM, lumbar paraspinal muscles; PA, phase angle.

The RDQ scores had a significant negative correlation with R of the LPM on both sides (left, r = −0.547, *p* = 0.035; right, r = −0.582, *p* = 0.023). ODI scores showed a similar negative correlation with R of the LPM on both sides (left, r = −0.523, *p* = 0.045; right, r = −0.581, *p* = 0.023). RDQ scores showed a positive correlation with PA of the right LPM (r = 0.521, *p* = 0.046) but no significant correlation with that of the left LPM (r = 0.428, *p* = 0.112) (Figure 3). No significant correlation was observed between the questionnaire scores and the other EIM data (*p* > 0.05).

## Discussion

This study applied the EIM technique to studying CLBP, which has not been reported previously. Its objective was to identify the difference in the EIM values of the LPMs between patients with CLBP and healthy individuals. The results showed that the R and Z were increased, X was similar, and PA was decreased in patients with CLBP relative to HCs. Significant correlations were found between the changed electrical properties and functional indexes of daily life in the patients; these underscore the importance of studying the electrical properties of the LPM.

### Electrical Impedance Myography Parameters Reflect the Status of Intracellular and Extracellular Matrices of Muscle Tissue

Electrical impedance myography can be used to assesses disease-induced changes to muscle's normal composition and architecture, including myocyte atrophy and loss, edema, reinnervation, and the deposition of endomysial connective tissue and fat (13). Skin, subcutaneous fat, and bone underlying the voltage electrodes do not significantly affect the results. The inherent resistivity of skeletal muscle is over an order of magnitude lower than that of fat and two orders of magnitude lower than that of bone, and together with the high detector impedance, these, therefore, have a minimal effect on the muscle impedance signatures (33). The impact of skin-subcutaneous fat layer thickness on EIM measurements had been previously reported by Tarulli et al. (34). They found that skin-subcutaneous fat layer thickness did not contribute substantially to the phase measured by linear EIM. The intracellular and extracellular matrices of the muscle tissue act as resistors, and any atrophy that reduces the CSA of muscle tissue increases the R value (35). On the other hand, changes in the X value are related to changes in the features of the cell membrane and layers of tissue (36). Coutinho et al. (37) suggested that changes in X were likely to be caused by changes in the features of the cellular membranes. In this study, the R and Z values increased significantly at 5 kHz in the CLBP group. However, no differences in the X and PA values were observed between the two groups. Because of the membrane permeability effects that occur at low frequencies, only a high-frequency current can flow through the intracellular compartment, and the current flow can be interrupted at 5 kHz. Our study showed that the R and Z values in the CLBP group were increased, and that the PA was decreased, as compared to the HC group. There was no significant difference in the X value at 50, 100, or 200 kHz. This was partly consistent with the results of the study by Ching et al. (21), who assessed the electrical properties of the LPM in patients with acute LBP and reported that the intracellular R value at 100 kHz was larger in these patients than in healthy participants.

The R and Z values that were elevated in the CLBP group indicated that the LPM might not have significantly atrophic changes or that changes were induced by localized edema of the muscle. Statland et al. performed a prospective study on 16 bilateral limb and trunk muscles in 35 genetically defined and clinically affected patients with facioscapulohumeral muscular dystrophy, using EIM at 50 and 100 kHz (18). They showed that the R and PA did not change, but that X showed differences. R did not increase in the patients with facioscapulohumeral muscular dystrophy. These results are in contrast to those of this study. The volume of the muscle has an impact on the EIM measurement (38) and localized oedema and inflammation which reduce the resistance often appeared in acute or subacute autoimmune conditions (25). CSA negatively correlated with the muscle's inherent impedance, and the decrease in the CSA score resulted in an increase in R (38). Sweeney et al. reported that the size and percentage change in the thickness of the lumbar multifidus muscle in patients with CLBP at the L4/L5 level did not differ between the two sides or from that in HCs (39). We also found no significant differences in EIM parameters between the two sides of the LPM in our patients (Figure 2). Thus, we concluded that the volumes of the LPM were bilaterally symmetrical in our patients. A previous study assessed EIM sensitivity to experimental inflammation induced by localized intramuscular injection of λ-carrageenan. Substantial reductions in phase and X in 5 kHz EIM values persisted at 48 and 72 h in the λ-carrageenan-treated group (40). However, changes in muscle composition can also affect the R and Z values. Increased fatty infiltration or connective tissue will have an impact on the measured electrical impedance (13).

The phenomenon of increased R and Z in the study may be due to extracellular remodeling, such as deposition of fat or and connective tissue. Most chronic myopathies are accompanied by substantial compositional changes in the muscle, including the deposition of fat and connective tissue (12). Goubert et al. (41) used MRI to evaluate differences in muscle structure and function in patients with RLBP, non-continuous CLBP, and continuous CLBP. They found that fat CSA and lean muscle fat index were significantly higher in the multifidus and erector spinae in cases with continuous CLBP than in those with non-continuous CLBP and recurrent LBP. Moreover, the fat area within the multifidus in patients with CLBP was larger than that in the controls (42). Capacitance, due to membrane structure and function, is a function of reactance and causes the current to lag behind the voltage, creating a phase shift or PA13 that is quantified as the angular transformation of the ratio of reactance to resistance (38). Based on these observations, the R value was increased and the X value was not changed in the CLBP group, indicating that the cellular membranes of the LPM in these patients might be undamaged, and that the number of cells was possibly similar to that in healthy individuals. This corroborates our finding that the peak value did not shift at different current frequencies.

To minimize the effect of muscle anatomy on impedance analysis, PA was used to describe the relationship between R and X (43). This evaluates the membrane oscillation properties of the muscle. In this study, the PA values were decreased in the CLBP group, which indicated that the membrane oscillation properties of the LPM were changed in patients with CLBP. Li et al. (44) suggested that the decrease in PA can be demonstrated by two representative patterns of the R and X association. The first pattern shows larger R values in the muscles of patients with CLBP than in the HCs and similar X values in the muscles of both groups across multiple frequencies. In the second pattern, the X values were smaller in the muscles of patients with CLBP than in the controls, whereas R remained similar in the muscles of both groups. In this study, the results demonstrated the first pattern. This indicated that the LPM in young adults with CLBP might have increased fatty infiltration, and the cellular membrane oscillation properties were decreased. However, the cellular membranes remained intact, and the number of cells did not change. Hu et al. have analyzed the correlation between muscle structures and electrical properties of the tibialis anterior in subacute stroke survivors (17). They found the decreased PA and elevated R and X in the affected tibialis anterior muscle of the subacute stroke survivors related to the lost muscle fibers. Therefore, the muscle fibers of LPM in the patients with CLBP in this study might also have been lost to some degree, with increased fatty infiltration and connective tissue formation. Alternatively, there were multiple types of pathological changes that contributed to the results. Nonetheless, this is an inference based on the results of this study and requires further research.

### Electrical Properties of the LPM Are Altered in Patients With CLBP at Different Current Frequencies

Multifrequency EIM is an extension of the linear EIM technique, which opens a previously unexplored dimension for EIM testing, potentially enhancing its ability to evaluate neuromuscular conditions (33). Multifrequency EIM overcomes the drawbacks of using a single frequency and provides a method for further assessment of the disease states of the muscle. In some muscle disease states, the frequency spectrum showing changes may differ (45). A previous study on R, X, and PA vs. frequency drew comparisons between healthy individuals and similarly aged patients with inclusion body myositis and found changes in the cellular structure due to the disease. However, the study reported that the disease created a shift in the peak phase to much higher frequencies (13). In this study, we investigated the electrical properties of the LPM of the two groups, measured at four different current frequencies, to explore whether there were results differed under different frequency conditions. The peak phase did not exhibit a significant shift at different current frequencies (Figure 2). This result indicates that the cellular structure of the LPM in patients with CLBP was not significantly altered as compared to that in the healthy individuals.

### Correlations Between Changes in Electrical Properties at a Current Frequency of 50 kHz and Disability in Daily Life in Patients With CLBP

The ODI is an effective method for measuring disability in patients with back pain presenting with varying degrees of severity and different causes. This index has stood the test of time, and it has been used in various clinical situations in most developed countries worldwide (20). On the other hand, the RDQ is a self-rated assessment of physical function in patients with back pain. It is a short and simple method, making it suitable for combining with other measures of function (e.g., work disability or psychological) in research settings. The ODI and RDQ have good content validity, construct validity, test–retest reliability, and responsiveness (30). These two methods are often used to assess the activities of daily life functions in patients with CLBP (46).

A standardized current frequency of 50 kHz is commonly used (12). In this study, we found that the ODI scores of the patients with CLBP correlated negatively with R, although both were increased in the CLBP group as compared to the HCs, at 50 kHz. Correlation analysis showed that RDQ scores correlated negatively with R, and positively with PA, whereas there was no significant correlation between X and RDQ scores in the CLBP group (Figure 3). These results were somewhat unexpected. The correlation plots indicated that a lower R was associated with worse disease, but patients had higher R values than did the HCs. This implied that the disease status of the LPM might not be as serious in the less-disabled patients. We considered two explanations for these results. One is that the patients with scores indicating greater disability might be at the “early stage” of CLBP; thus, the structural changes in the muscle were less than in those who had lower scores. Patients with CLBP lose muscle fibers or have fatty infiltration and increased connective tissue in the LPM, which tends to generate compensatory adaptation strategies for daily living (47), preventing significant disability in these persons. Since self-evaluated questionnaires and the electrical properties of the LPM are independent phenomena, a negative correlation between these parameters might be caused by multiple factors. Another explanation is that the pathological relationship between function and the LPM electrical properties was observed in only a small number of patients. Changes in the LPM physiology or pathology may be due to a combination of factors (48). Given the limited sample size and population of this study, this result might be not applicable to all patients with CLBP. Nonetheless, the results of correlation analysis in the study offered basic evidence that the EIM technique can be applied for electrical property measurements in CLBP. As an objective and easily operable measurement method, EIM should be widely applied for the diagnosis of CLBP in future.

### Limitations

This study also had some limitations. First, the small sample size could have limited these findings and might present pathological characteristics of the LPM in only a part of this patient population. Second, the age of the participants included in the study was a limitation. Measurements were taken only for the LPM in young adults, thus limiting the generalizability of the results. Difference in the age and sex of the patients with CLBP may consequently present different structural or functional status in their LPMs (49, 50). Third, the anatomical structure of the lumbar crest did not permit the measurement of the EIM parameters in the transverse direction; hence, the anisotropy ratios of each variable were not calculated. Muscle anisotropy represents the degree of columnar order in the arrangement of the fibers (51); however, the arrangement of the LPM fibers in CLBP was not assessed in this study. Fourth, the location of the pain in the low back might result in different electrical impedance values. Since some of the patients in the study could not state the location of their pain accurately, we did not distinguish patients into unilateral and bilateral pain groups. This should be investigated in future.

### Conclusions

The electrical properties of the bilateral LPMs differed between the patients with CLBP and HCs, regardless of the current frequency used. The R and Z values were elevated, PA values were decreased, and no significant changes occurred in the X values in young adults with CLBP, as compared to HCs. This may indicate that the LPM of the patients with CLBP has fewer muscle fibers with increased fatty infiltration or connective tissue, and that the cellular membranes are not damaged, but that their oscillation properties are altered. The altered electrical properties of the LPM correlated with disability in daily life. This suggests that exploring the electrical properties of the LPM in patients with CLBP is meaningful and can contribute to discovering the pathological characteristics of the muscle composition. EIM, as a novel technique for assessing CLBP, should have widely applications in future.

## Data Availability Statement

The datasets analyzed during the current study are available from the corresponding authors upon reasonable request.

## Ethics Statement

The studies involving human participants were reviewed and approved by the Human Subjects Ethics Subcommittee of the First Affiliated Hospital of Sun Yat-sen University Number: 2020425. The patients/participants provided their written informed consent to participate in this study. Written informed consent was obtained from the individual(s) for the publication of any potentially identifiable images or data included in this article.

## Author Contributions

HW, LL, and CW conceived and designed the study. HW, JZ, and ZF performed the experiments. HW, WL, and QY analyzed the data and wrote the paper. WL, QY, LL, and CW reviewed and edited the manuscript. All authors have read and approved the manuscript.

## Funding

This work was supported by the National Natural Science Foundation of China (Grant Numbers 81772434, 82172532, 8200375, and 31771016), the Development Center for Medical Science and Technology, National Health Commission of the People's Republic of China (Grant Number DCMST-NHC-2019-AHT-01), and the Guangdong Basic and Applied Basic Research Foundation (Grant Number 2020A1515011356).

## Conflict of Interest

The authors declare that the research was conducted in the absence of any commercial or financial relationships that could be construed as a potential conflict of interest.

## Publisher's Note

All claims expressed in this article are solely those of the authors and do not necessarily represent those of their affiliated organizations, or those of the publisher, the editors and the reviewers. Any product that may be evaluated in this article, or claim that may be made by its manufacturer, is not guaranteed or endorsed by the publisher.
